# Supplementary material for: Inhibition of Metalloproteinases Extends Longevity and Function of In Vitro Human iPSC-Derived Skeletal Muscle
Source: Biomedicines. 2024 Apr 12;12(4):856. doi: 10.3390/biomedicines12040856 (PMC11047953; doi:10.3390/biomedicines12040856)
Supplement: Supplementary file 1 [file biomedicines-12-00856-s001.zip › biomedicines-2912439-supplementary.pdf]

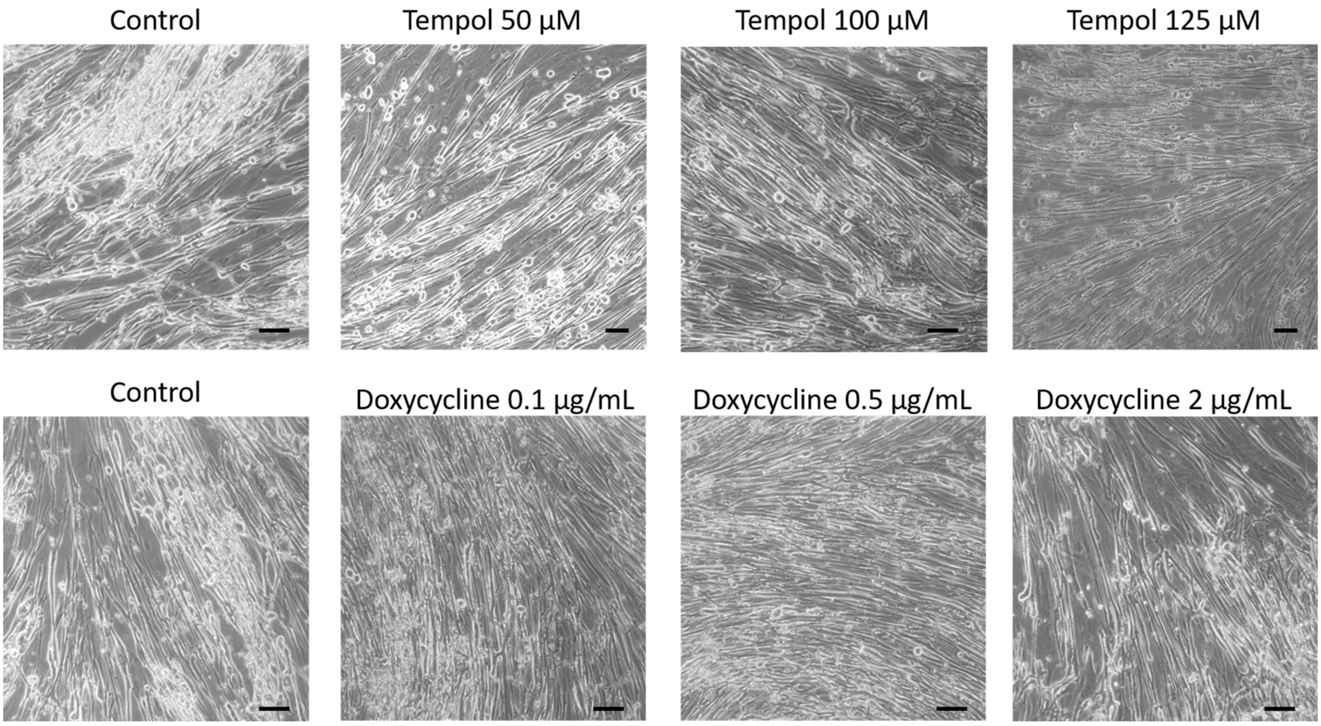

Figure S1: Concentration optimization for Tempol and doxycycline doses in in vitro conditions. Representative images shown on Day 30 upon switching to differentiation medium. Tempol at 100 μM enabled better myotube preservation than 50 μM. Further increases in the dosage have a negative effect. Similarly, doxycycline at 0.5 μg/mL gave the highest number of myotube preservation. Scale = 100 μm.
